# Therapeutic targeting of the cGAS-STING pathway in human disease

**DOI:** 10.1172/JCI204547

**Published:** 2026-07-01

**Authors:** Akanksha S. Mahajan, Connor M. Forsyth, Cao Dai Phung, Xinhe Shen, Rachel Jarvis, Alexander H. Stegh

**Affiliations:** 1 Taylor Family Department of Neurosurgery, The Brain Tumor Center, Alvin J. Siteman Comprehensive Cancer Center, Washington University School of Medicine, Washington University, St. Louis, Missouri, USA.; 2 Interdisciplinary Biological Sciences Graduate Program, International Institute for Nanotechnology, Northwestern University, Evanston, Illinois, USA.

## Abstract

The cyclic GMP-AMP synthase–stimulator of interferon genes (cGAS-STING) pathway is a central regulator of innate immunity that links cytosolic DNA sensing to type I IFN and inflammatory responses. While initially viewed as a uniformly beneficial antiviral and antitumor signaling axis, emerging evidence reveals that cGAS-STING functions as a context-dependent immune rheostat whose impact is dictated by signal magnitude, timing, cellular origin, subcellular localization of signaling components, and tissue context. These parameters explain why pathway activation can promote tumor rejection, vaccine efficacy, and host defense in some settings yet drive immune suppression, metastasis, neuroinflammation, or autoinflammatory disease in others. In this Review, we synthesize mechanistic and clinical insights across agonist and antagonist strategies targeting the cGAS-STING pathway in cancer, infectious disease, neurodegeneration, and interferonopathies. We highlight why first-generation STING agonists have underperformed clinically and how next-generation delivery systems and cGAS-directed approaches may overcome these limitations. We propose a disease-centric framework that integrates spatial delivery, dosing architecture, and pharmacodynamic biomarker discovery to enable rational modulation of cGAS-STING, repositioning the pathway as a tunable immunologic control node for precision therapy rather than a binary on/off switch.

## Introduction

The discovery of the cyclic GMP-AMP synthase–stimulator of interferon genes (cGAS-STING) pathway reshaped our understanding of innate immune sensing, revealing how cytosolic DNA detection links innate and adaptive immunity through 2′3′-cyclic GMP-AMP–mediated (cGAMP-mediated) activation of STING and type I IFN signaling (1, 2). Originally characterized as a cytosolic surveillance system that senses foreign DNA and triggers type I IFN responses, cGAS-STING rapidly emerged as a central node linking innate and adaptive immunity (3) required for antiviral defense and tumor immune surveillance (4). The same pathway that protects against pathogens and malignancy can, when dysregulated, drive autoinflammatory syndromes, neurodegeneration, chronic tissue damage, and even tumor progression.

Therapeutic development has largely followed a simplified paradigm, activating cGAS-STING to enhance antitumor immunity and inhibiting it to treat inflammatory disease, but clinical outcomes have exposed limitations to this approach. Despite extensive clinical evaluation, STING agonists have demonstrated only modest antitumor activity, highlighting the context-dependent biology of this pathway. Genetic and pharmacologic studies now demonstrate that cGAS-STING signaling does not encode a fixed outcome; instead, its effects depend on signal magnitude and duration, cellular and subcellular localization of pathway components, and tissue context, which together determine whether signaling promotes immunity, tolerance, or pathology.

In this Review, we summarize current strategies to activate or inhibit cGAS-STING signaling, integrating mechanistic insight with clinical experience to explain why agonist and antagonist approaches succeeded — or failed — depending on disease context, delivery strategy, and timing of drug administration. We highlight advances in drug design, spatially controlled delivery, and the discovery of pharmacodynamic biomarkers and propose a disease-centric framework to guide rational therapeutic targeting of this pathway across cancer, infection, neurodegeneration, and autoinflammatory disease.

## Molecular architecture of the cGAS-STING signaling axis

cGAS is a cytosolic DNA sensor that recognizes double-stranded (ds)DNA irrespective of its sequence or origin and detects both microbial DNA and endogenous DNA derived from the nucleus or mitochondria (1, 5). cGAS discriminates self from non-self by macromolecular context: nucleosome-bound DNA binds cGAS yet suppresses its catalytic activity, demonstrating that DNA structure governs cGAS activation (5–7). DNA binding drives the formation of 2:2 cGAS:DNA complexes and cooperative ladder-like assemblies (8) that undergo phase separation into condensates to amplify the synthesis of the cyclic dinucleotide (CDN) cGAMP (9). cGAMP activates the ER adaptor STING, triggering conformational changes, its oligomerization (10, 11), and recruitment of TANK binding kinase 1 (TBK1) (10, 12–15). TBK1 phosphorylates the STING C-terminal tail to recruit interferon regulatory factor 3 (IRF3) for type I IFN induction (13) and promotes TNF receptor associated factor 6 (TRAF6) activation to engage NF-κB signaling (14, 16). Together, this spatially organized, multistep assembly converts cytosolic DNA recognition into a coordinated transcriptional program that links DNA sensing to broad immune activation against viruses and tumors (see Table 1 describing the major upstream regulators of cGAS and STING).

Notably, cGAS-STING pathway competency is frequently altered. Multiple tumor types exhibit reduced expression of cGAS, STING, or downstream signaling mediators; in some cases, downregulation of pathway components is associated with promoter methylation, repressive chromatin states, or broader transcriptional silencing programs that facilitate immune evasion (17–19). Furthermore, attenuation of the cGAS-STING pathway can coexist with chromosomal instability and cytosolic DNA stress, suggesting that tumor evolution may select for functional genomic states that uncouple DNA damage from innate immune sensing (20, 21). In other settings, retained or rewired cGAS-STING activity can contribute to inflammatory adaptation, stromal remodeling, metastatic dissemination, or therapeutic resistance (22–24).

## Therapeutic activation of the cGAS-STING pathway

**Figure 1:**
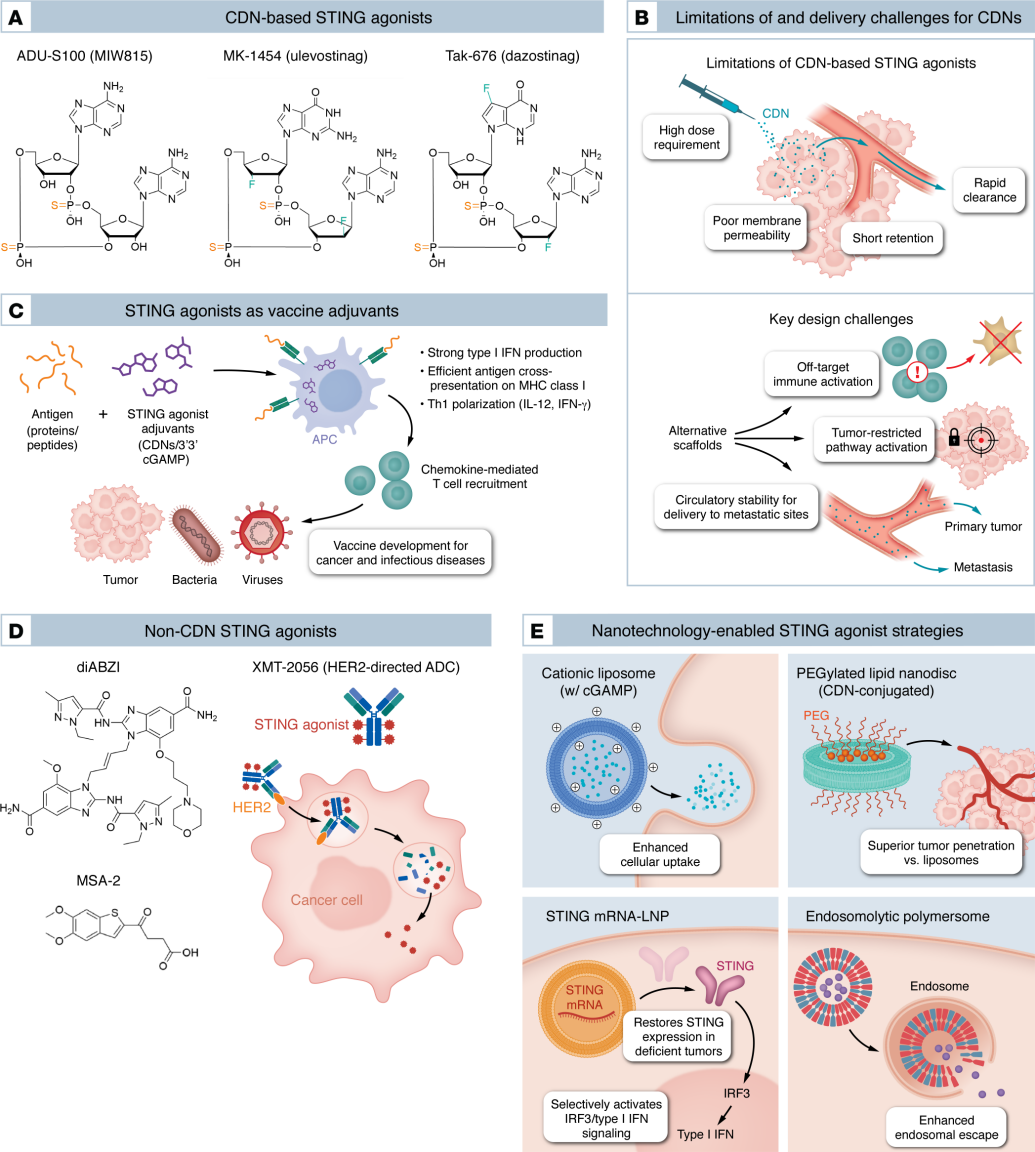
STING agonists in cancer immunotherapy and infectious disease: mechanisms, molecular classes, and emerging clinical strategies. (**A**) Representative cyclic dinucleotide (CDN) STING agonists in clinical development include ADU-S100 (MIW815), dazostinag (TAK-676), and MK-1454 (ulevostinag). Structural modifications (e.g., phosphorothioate linkages and halogen substitutions) are highlighted that improve metabolic stability, potency, and in some cases, systemic delivery following intratumoral or intravenous administration. (**B**) Key limitations of CDNs include rapid clearance, poor membrane permeability, high dose requirements, and limited tumor retention. (**C**) STING agonists can act as vaccine adjuvants. CDNs and 3′3′-cGAMP enhance antigen presentation by antigen-presenting cells, drive robust type I IFN production, promote Th1 polarization, and improve CD8^+^ T cell priming and chemokine-mediated effector recruitment, enabling dose-sparing immunity against viral pathogens, cancer antigens, and bacterial infections. (**D**) Non-CDN small molecule STING agonists (e.g., amidobenzimidazole [ABZI], diABZI, and MSA-2) and antibody drug conjugates (ADCs) for cancer-selective targeting (e.g., XMT-2056) illustrate orally or intravenously bioavailable agents with improved pharmacokinetics, cancer selectivity, and broader human STING allele coverage compared with CDNs. (**E**) Nanotechnology-enabled STING agonist approaches may overcome delivery limitations by improving tissue targeting, cellular uptake, pharmacokinetics, and therapeutic index, supporting durable antitumor immune responses while mitigating systemic toxicity.

**Figure 2:**
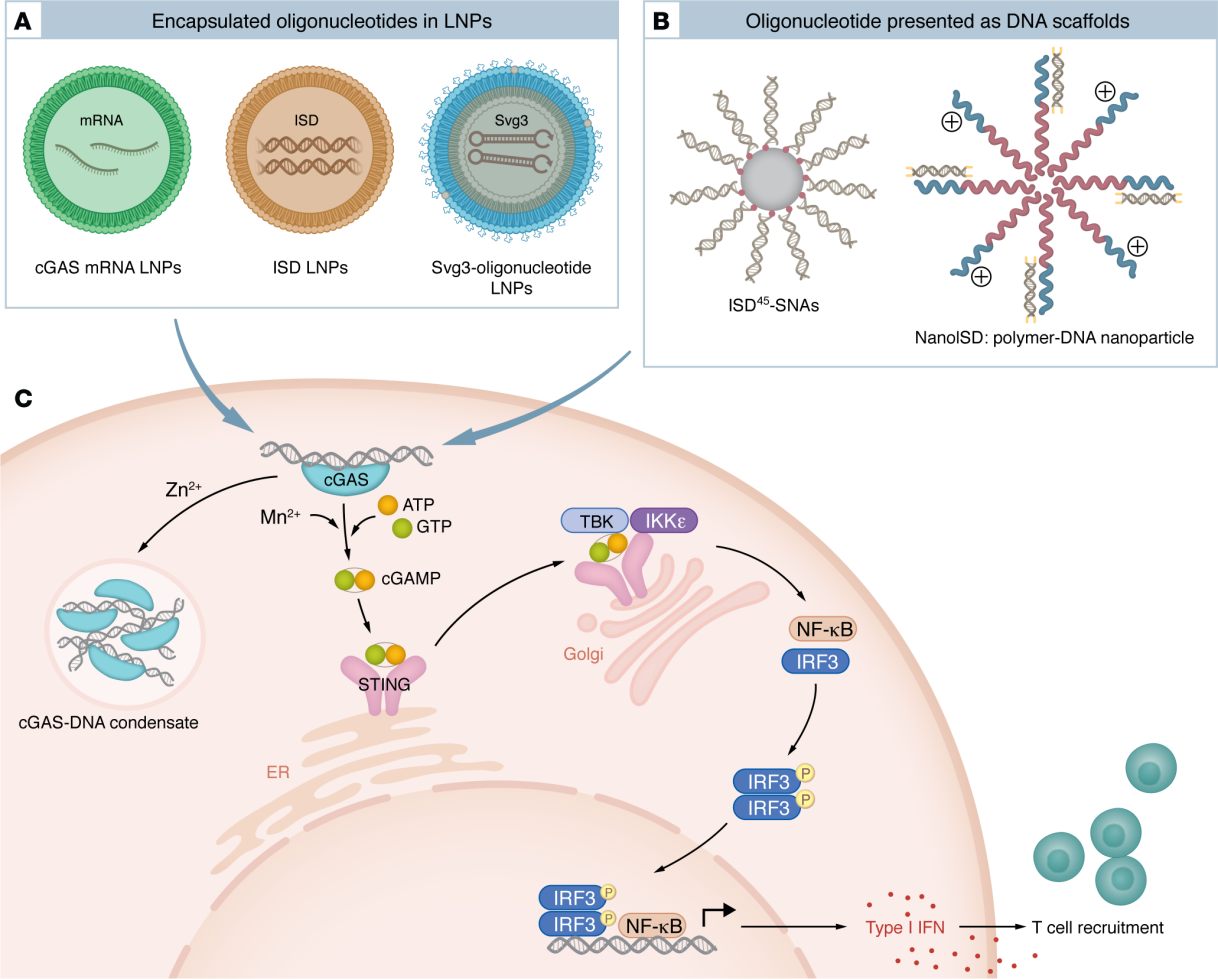
cGAS agonist delivery platforms and intracellular signaling outcomes. (**A**) Encapsulated oligonucleotide approaches using lipid nanoparticles (LNPs) include cGAS mRNA LNPs to drive intracellular cGAS expression, interferon-stimulatory dsDNA (ISD) LNPs, and synthetic oligonucleotide cGAS agonists, enabling cytosolic access while improving stability and biodistribution. (**B**) Architectures with ISD presented as higher-order scaffolds include spherical nucleic acids (ISD^45^-SNAs) and polymer-DNA nanoparticles (NanoISD), which enhance cellular uptake, protect cargo from degradation, and promote efficient engagement of cGAS. (**C**) Cytosolic dsDNA or DNA scaffolds bind and activate cGAS, a process potentiated by divalent cations such as Mn^2+^, leading to the conversion of ATP/GTP into the second messenger cGAMP. In cGAS–DNA phase-separated condensates, Zn^2+^ acts as a protein condensate enhancer that stabilizes signaling hubs and amplifies pathway output. cGAMP produced by cGAS (with Mn^2+^ as a critical cofactor) subsequently activates STING at the ER, driving its trafficking to the Golgi and activation of TBK1 and IKKε, resulting in IRF3 phosphorylation and NF-κB signaling. These pathways culminate in robust type I IFN production and transcription of pro-inflammatory cytokines, including TNF-α, IL-6, and IL-1β.

Pro-inflammatory functions of the cGAS-STING pathway provide a strong therapeutic rationale for pathway agonism in two principal clinical settings. First, in cancer immunotherapy, activation of the cGAS-STING pathway converts immunologically “cold” tumors — characterized by low mutational burden, sparse T cell infiltration, and tolerogenic myeloid populations — into “hot,” inflamed tumors capable of responding to checkpoint inhibition (25–27). Second, in infectious disease, cGAS-STING activation and the resulting type I IFN response are designed to constrain viral replication and limit bacterial dissemination (28, 29) (Figure 1, Figure 2, and Table 2).

### STING agonists for cancer immunotherapy.

Early translational efforts focused on synthetic CDN-based STING agonists, such as ADU-S100 (30–36) and MK-1454 (37). Across preclinical models, these agents induced dendritic cell (DC) priming, CD8^+^ T cell activation, tumor regression, and, in some settings, durable immune memory, supporting their clinical development. Clinical studies with ADU-S100 (NCT03172936, NCT02675439) (38, 39) and MK-1454 (NCT03010176) (40) showed that intratumoral STING activation is feasible, pharmacodynamically active, and generally manageable in patients with advanced solid tumors or lymphomas. However, ADU-S100 monotherapy efficacy was limited, and similarly, MK-1454 produced no confirmed objective responses in heavily pretreated patients. When combined with the immune checkpoint inhibitor pembrolizumab (NCT04220866), MK-1454 improved progression-free survival in recurrent/metastatic head and neck squamous cell carcinoma, with durable responses limited to the combination arm, supporting STING agonists as rational partners for immunotherapy combinations (40).

Dazostinag (TAK-676) is a systemically deliverable synthetic CDN developed by Takeda to address the pharmacokinetic and delivery limitations of earlier STING agonists. In preclinical models, systemic TAK-676 treatment activated innate and adaptive immunity and produced durable tumor regressions with immunologic memory (41). TAK-676 displayed favorable tolerability, dose-proportional pharmacokinetics, and preferential tumor exposure, supporting advancement into early-phase clinical trials (41). In heavily pretreated patients with advanced solid tumors, TAK-676, in combination with pembrolizumab (NCT04420884), demonstrated manageable safety and achieved durable disease stabilization despite limited objective responses (42).

Thus, CDN-based STING agonists, ranging from intratumoral agents to systemically deliverable compounds, can engage type I IFN–dependent DC priming and CD8^+^ T cell activation. While this biology drives robust tumor regression and durable immune memory in preclinical models, clinical responses to CDNs as monotherapy have been modest, underscoring a persistent gap between mechanistic promise and therapeutic efficacy.

### STING agonists as vaccine adjuvants.

In cancer and infectious disease vaccines, cGAS-STING agonists are emerging as next-generation adjuvants that link innate sensing to adaptive immunity. Compared with TLR agonists such as CpG or poly(I:C), STING activation induces stronger type I IFN signaling, antigen crosspresentation, Th1 polarization, antigen-specific CD8^+^ T cell expansion, and CXCL9/CXCL10-mediated recruitment of CXCR3^+^ effector T cells (43–46). CDN adjuvants — including c-di-GMP, c-di-AMP, and 3′,3′-cGAMP — enhanced antibody titers, CD8^+^ T cell priming, and immune memory, and STINGVAX, a synthetic CDN incorporated into a GM-CSF–secreting whole-cell tumor vaccine platform, showed antitumor activity and synergy with programmed cell death 1 (PD-1) blockade, including in poorly immunogenic models (31, 47–49).

In infectious disease models, STING agonists demonstrated dose-sparing and mucosal adjuvant activity across influenza, HIV, hepatitis B, herpes simplex virus, and SARS-CoV-2 (46, 50–55). Notably, intranasal cGAMP-based formulations improved protection against influenza, including dose-sparing immunity and crossprotection against heterosubtypic strains (50, 51). Collectively, these data support STING agonists as broadly applicable vaccine adjuvants, particularly where robust cellular immunity, mucosal protection, and durable dose-sparing responses are needed.

### Challenges in translating first-generation CDN-based STING agonists.

Owing to their small size, hydrophilicity, and poor membrane permeability, CDNs exhibit rapid systemic clearance, inefficient cytosolic delivery, and limited tumor retention (56, 57). Consequently, achieving adequate intratumoral exposure often requires high doses or direct intratumoral injection — an approach restricted to anatomically accessible lesions. Even with local delivery, CDNs rapidly diffuse from the tumor microenvironment (TME), resulting in transient STING activation. For example, ADU-S100 exhibits a terminal plasma half-life of approximately 24 minutes following intratumoral injection in patients with solid tumors or lymphoma (39). This rapid dissemination primarily produces short-lived cytokine surges that do not effectively reprogram the immune response.

An additional challenge in agonist development is the extensive polymorphism of human STING. The major STING haplotypes differ in their responsiveness to endogenous CDNs, with some variants displaying hypomorphic behavior (33, 58, 59). Synthetic agonists such as ADU-S100 and the non-CDN ABZI class, discussed below, were developed in part to overcome this limitation by engaging the conserved STING dimer interface to stabilize the receptor’s closed, active conformation. For ABZI and related non-nucleotide agonists, this mode of STING engagement supports potent human STING activation (60) and, in related compounds such as SHR1032, can preserve activity across common human STING alleles, including R232, H232, and HAQ, though potency remains allele dependent (61). Nonetheless, broad allele activity should not be equated with uniform potency or complete functional rescue of hypomorphic, severely defective, or true null STING variants. Therefore, STING genotyping may remain an important consideration for patient selection, biomarker development, and next-generation agonist design.

Tumors with high chromosomal instability (CIN) often exhibit a rewired STING pathway, in which chronic cytosolic DNA exposure favors noncanonical NF-κB signaling over protective type I IFN responses, thereby promoting metastasis and immune evasion (20, 22, 62). In addition, loss-of-function mutations or epigenetic silencing of *STING1* or *MB21D1* render some tumors functionally STING deficient and unresponsive to CDNs (63). Accordingly, therapeutic benefit most likely occurs in tumors with intact STING signaling, high cGAS expression, and low baseline IFN activity, whereas tumors with constitutive IFN signatures, CIN-driven chronic activation, or pathway silencing are unlikely to respond. Integrating biomarkers such as STING/cGAS expression, IFN-stimulated gene (ISG) signatures, and CIN status into trial design is therefore critical for distinguishing productive immune priming from immune tolerance reinforcement.

### Next-generation STING agonists: non-CDN small molecule activators.

The limitations of CDN-based STING agonists have motivated the development of systemically deliverable agents built on alternative chemical scaffolds, alongside strategies aimed at overcoming three central challenges: preventing cytokine release from off-target immune activation, achieving tumor-restricted pathway engagement despite systemic administration, and preserving sufficient circulatory stability to enable drug distribution to distant metastatic sites.

Several non-CDN STING agonist classes, including benzothiophenes, ABZI compounds, and diABZI analogs, have been developed through screening and structure-guided design, with improved potency, systemic stability, and human STING engagement over first-generation CDNs. Ramanjulu et al. identified ABZI agonists active across common human STING haplotypes; a lead compound showed favorable intravenous pharmacokinetics and CD8^+^ T cell–dependent durable tumor regression in mice (60). Pan et al. described MSA-2, an orally bioavailable small molecule STING agonist that stabilizes active STING, induces TBK1/IRF3 signaling, promotes durable antitumor immunity, and synergizes with PD-1 blockade, partly through enhanced activity in acidic TMEs (64).

To improve spatial control and limit systemic toxicity, ADCs were developed that couple STING agonists to tumor-targeting antibodies, thereby restricting pathway activation to the TME. XMT-2056, a HER2-directed STING agonist ADC developed by Mersana Therapeutics, enabled antigen-dependent uptake into tumor cells and Fcγ receptor–mediated delivery to tumor-resident myeloid cells, resulting in robust type I IFN signaling, immune cell infiltration, and tumor regression across HER2-high and HER2-low models (65). Compared with a free agonist, XMT-2056 demonstrated enhanced efficacy and reduced systemic inflammation, and it synergized with HER2-targeted therapies and PD-1 blockade (65).

In addition to direct agonists, increasing attention has focused on indirect modulation of the cGAS-STING pathway by inhibiting endogenous negative regulators. ENPP1 represents a distinct regulatory node: as the preeminent extracellular cGAMP hydrolase, ENPP1 limits paracrine STING activation and contributes to immune suppression in the TME. Accordingly, ENPP1 inhibitors have gained traction as a strategy to preserve extracellular cGAMP, thereby extending STING signaling to neighboring host cells, and in some contexts, reducing immunosuppressive nucleotide metabolism (66–68). This approach differs from direct STING agonism in that it amplifies the endogenous pathway input rather than directly activating the receptor.

### Nanotechnology-enabled STING agonists.

To address the limited membrane permeability of STING agonists, nanotechnology-based platforms have been developed to enable more effective STING immunotherapy by improving uptake, cytosolic delivery, tumor retention, and spatial control of pathway activation, with liposomal and polymer-based carriers enhancing intracellular access and endosomal escape (57, 69–72). Notable nanotherapeutic platforms include endosomolytic polymersomes (71) and poly(β-amino ester) nanoparticles covalently conjugated to a CDN via a cathepsin-cleavable linker (73). In addition, PEGylated lipid nanodiscs conjugated with CDNs demonstrated superior tumor diffusion relative to liposomes after systemic dosing (57). Enhanced CDN spatial distribution facilitated the colocalization of dying tumor cells and DCs, promoting effective antigen crosspresentation and T cell priming. A single intravenous dose induced tumor rejection and durable immune memory in multiple models, illustrating how nanoparticle geometry and deformability can shape immunologic outcomes (57).

Coordination chemistry provided a means to directly potentiate cGAS-STING signaling via metal-ion cofactors. Manganese (Mn^2+^), identified as a potent enhancer of STING activation across human haplotypes, was coassembled with CDNs to form self-forming coordination nanoparticles (CNPs), enabling robust antitumor immunity at minute doses following either intratumoral or systemic administration (72). Similarly, Zn^2+^-based nanoscale coordination polymers encapsulating cyclic di-AMP (ZnCDA) prolonged CDN circulation, enhanced tumor accumulation, and achieved potent single-dose efficacy across diverse models (74). Mechanistically, ZnCDA disrupted tumor vasculature and preferentially targeted tumor-associated macrophages, augmenting antigen processing and T cell priming (74). These platforms illustrate how rational manipulation of metal-ion cofactors can integrate signal amplification with delivery, defining a new class of “metalloimmunotherapy.”

Finally, synthetic pathway reconstitution strategies can address tumors with low or absent endogenous STING expression by delivering *STING1* mRNA via lipid nanoparticles (LNPs). This approach selectively activated IRF3/type I IFN signaling while minimizing NF-κB–driven protumor inflammation and functioned independently of tumor-intrinsic STING status (75). By shifting STING therapy from agonist delivery to programmable restoration of pathway components, this strategy may overcome epigenetic silencing or mutational inactivation of the cGAS-STING axis.

Across these platforms, three unifying principles emerge. First, intracellular access is critical: endosomal escape and controlled cytosolic delivery determine whether STING agonists efficiently engage intracellular STING and help shape the magnitude, duration, and immunologic quality of the resulting response. Second, the spatial distribution of pathway activation governs the coupling of innate and adaptive immunity, with nanoparticle size, shape, and deformability controlling tissue penetration and colocalization with APCs. Third, controlling the extent of signal amplification is critical: metal-ion coordination, linker chemistry, and pathway reconstitution strategies modulate signaling amplitude and bias (IRF3 versus NF-κB), thereby shaping efficacy and tolerability. Together, these advances position nanoarchitectures not simply as passive formulation platforms but as active regulators of innate immune signaling.

### Discontinuation of STING agonist programs.

Where available, the reasons for discontinuation of early STING-directed programs are instructive. Development of XMT-2056 (NCT04486378) was interrupted in 2023 after the emergence of a treatment-related grade 5 adverse event, which prompted voluntary study suspension and an FDA clinical hold. Although the clinical hold was subsequently lifted after protocol adjustments, this example underscores the challenge of balancing innate immune potency with safety and tolerability. By contrast, the discontinuation of ADU-S100 appeared to have been driven largely by its limited clinical efficacy; early-phase studies reported modest single-agent antitumor activity despite evidence of immune activation, and sponsor communications indicated portfolio discontinuation based on the clinical data generated to date. Together, these examples suggest that attrition in the STING agonist field reflects distinct but recurring therapeutic challenges, including inadequate efficacy, restrictive drug distribution, and undesirable inflammatory toxicity, rather than the inadequacy of cGAS-STING as a therapeutic target.

### Activation of cGAS: mechanistic rationale and advantages.

Direct cGAS engagement offers several advantages over STING agonism: it is largely STING variant agnostic, exploits enzymatic signal amplification through endogenous cGAMP production, remains subject to physiologic feedback regulation, and may synergize with tumor-derived cytosolic DNA generated by genomic instability. Early approaches using naked dsDNA were limited by poor uptake, nuclease degradation, and lack of tissue specificity (76–78), prompting the development of engineered delivery platforms.

Nanotechnological systems enable more efficient delivery of cytosolic DNA. Spherical nucleic acids (SNAs), composed of radially oriented oligonucleotides on nanoparticle cores, enhance nuclease resistance, scavenger receptor–mediated uptake, and multivalent cGAS engagement (79, 80). In glioblastoma models, intratumorally or intranasally delivered SNAs conjugated with 45 bp interferon-stimulatory dsDNA (ISD^45^-SNAs) preferentially targeted tumor-associated macrophages, activated cGAS-STING signaling, reprogrammed local and cervical lymph node immunity, induced tumor regression, and synergized with checkpoint blockade (81). Complementary platforms include NanoISD, which packaged phosphorothioate-capped 95 bp dsDNA into endosomolytic polymer complexes to improve cytosolic delivery and intratumoral retention, thereby promoting type I IFN signaling, DC maturation, TME reprogramming, tumor control, and checkpoint responsiveness (82).

LNP-based strategies further extend cGAS activation to vaccine design. Svg3-adjuvanted LNP vaccines codelivering cGAS agonists with peptide or mRNA antigens localized to lymph node APCs, enhanced antigen presentation and T cell priming, reduced regulatory T cells, and improved tumor control, particularly with anti–PD-1 (83). Similarly, LNP delivery of engineered *MB21D1* mRNA promotes sustained STING-dependent type I IFN signaling, APC maturation, antigen crosspresentation, and enhanced humoral and cellular immunity, demonstrating antitumor activity as a standalone or vaccine-adjuvant approach (84, 85). However, mRNA- and oligonucleotide-based strategies remain constrained by delivery vehicles, parenteral administration, cold-chain requirements, and transient expression.

Collectively, these studies show that the major barrier to pharmacologic cGAS engagement is not the immunostimulatory capacity of DNA, but rather the efficient delivery of intact ligands to cytosolic cGAS. This hurdle is exacerbated by TREX1, the dominant cytosolic 3′–5′ exonuclease, which degrades dsDNA, thereby limiting cGAS activation by reducing ligand availability. Consequently, TREX1 has emerged as a candidate innate immune checkpoint. Preclinical studies suggested that TREX1 inhibition (analogous to targeting the cGAMP-degrading enzyme ENPP1 upstream of STING) could enhance endogenous cGAS-STING signaling and antitumor immunity (86).

## Inhibition of the cGAS-STING pathway

**Figure 3:**
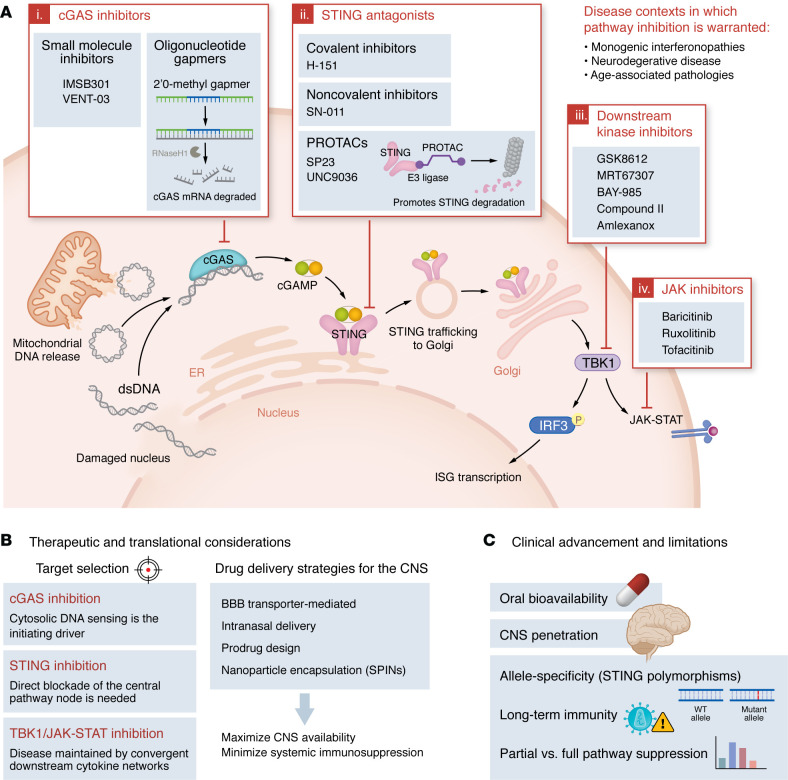
Disease contexts requiring cGAS-STING inhibition, molecular targets, and therapeutic considerations. (**A**) Disease contexts in which pathway inhibition is warranted include monogenic interferonopathies caused by gain-of-function mutations leading to lifelong pathway overactivation; neurodegenerative diseases characterized by mitochondrial dysfunction, impaired autophagy, and cytosolic release of self-DNA; and age-associated pathologies, where chronic, low-level cGAS-STING activation contributes to inflammaging, cardiovascular disease, and metabolic dysfunction. Therapeutic strategies targeting cGAS-STING signaling cascade include (i) cGAS inhibitors, such as small molecules and oligonucleotide gapmers, which block aberrant DNA sensing upstream; (ii) STING antagonists, including covalent and noncovalent inhibitors as well as proteolysis targeting chimera–based (PROTAC-based) degraders, which prevent STING activation or ER-to-Golgi trafficking; (iii) downstream kinase inhibitors, targeting TBK1 or associated signaling complexes to block IRF3 activation; and (iv) JAK inhibitors, which suppress interferon receptor–mediated JAK/STAT signaling and ISG expression. (**B**) Therapeutic and translational considerations. (Left) Indications for inhibitors targeting cGAS versus STING versus downstream signaling. (Right) Strategies to achieve effective central nervous system (CNS) delivery — including blood-brain barrier (BBB) transporter–mediated uptake, intranasal administration, prodrug design, and nanoparticle encapsulation — maximize CNS bioavailability while minimizing systemic immune suppression. SPINs, STING pathway inhibiting nanoparticles. (**C**) Clinical advancement challenges include oral bioavailability, CNS penetration, allele-specific responses driven by STING polymorphisms, long-term immune consequences, and the need to balance partial versus complete pathway suppression.

Pathogenic cGAS-STING activation drives chronic type I IFN signaling and inflammatory pathology across diverse human diseases (87, 88). Genetic studies support therapeutic antagonism, as cGAS or STING deficiency protects mice in models of neurodegeneration, pancreatitis, macular degeneration, ischemic injury, hepatic inflammation, and arthritis (89). Three major contexts justify pathway inhibition: monogenic interferonopathies caused by DNA metabolism defects or gain-of-function STING mutations; neurodegenerative diseases linked to mitochondrial dysfunction, impaired autophagy, and cytosolic DNA release; and age-associated inflammatory disorders driven by damaged organelles, cellular debris, and genomic instability. These settings require distinct strategies (Figure 3): near-complete but infection-sparing suppression in interferonopathies, cell type–selective inhibition in neurodegeneration, and intermittent or node-specific pathway modulation in age-associated disease.

### Therapeutic targeting of cGAS: silencing the alarm.

Most cGAS-STING inhibition efforts have focused on small molecules that block cGAS enzymatic activity (Table 3). Chemically modified 2′-O-methyl gapmer oligonucleotides can inhibit cGAS in a sequence-dependent manner, using terminal modifications to enhance stability and uptake while the DNA “gap” engages DNA-sensing receptors without productive activation (90). Several oral cGAS inhibitors are now in clinical development, including ImmuneSensor’s IMSB301, in phase I testing for inflammatory and autoimmune diseases, and Ventus Therapeutics’ VENT-03, the first cGAS inhibitor to complete a first-in-human phase I study, with potential applications in autoimmune, cardiometabolic, and age-associated inflammatory disorders (91).

cGAS activity can also be modulated by targeting its biophysical activation state. A novel class of protein condensation inhibitors binds an allosteric site near the activation loop to disrupt the phase-separated protein: DNA assemblies required for cGAS activation, demonstrating in vivo efficacy in a cerulein-induced acute pancreatitis model (92). These findings establish higher-order cGAS condensation as a druggable regulatory mechanism and expand the therapeutic landscape beyond traditional orthosteric inhibition.

### STING protein–targeted inhibitors.

Direct pharmacologic inhibition of STING has emerged as a compelling strategy to attenuate pathological type I IFN signaling in autoinflammatory and IFN-driven diseases (Table 3). Small molecule STING inhibitors act by binding the STING protein itself and preventing its activation, intracellular trafficking, or downstream signal propagation, through the stabilization of inactive conformations, blockade of cGAMP binding, or disruption of STING–TBK1 complex formation (93, 94). H-151 represents a prototypical covalent STING antagonist that irreversibly modifies cysteine residues (Cys88 and Cys91) within the ligand-binding domain, thereby locking STING in an inactive conformation incapable of recruiting or activating TBK1. In murine models of TREX1-deficient autoimmunity, early or therapeutic administration of H-151 prevented disease onset and reversed established inflammation by normalizing ISG expression, ameliorating multiorgan pathology, and restoring survival without overt susceptibility to routine microbial challenges under specific pathogen–free conditions (94, 95). However, therapeutic inhibition may be complicated by gain-of-function STING variants, such as N154S and V155M, which drive ligand-independent STING activation in STING-associated vasculopathy with onset in infancy (SAVI) (96, 97). These variants highlight the need for inhibitors that suppress constitutive STING trafficking/palmitoylation or promote its degradation, rather than relying solely on cGAMP blockade. In addition, irreversible and near-complete pathway inhibition raises concerns about long-term antiviral competence, particularly against latent herpesviruses prevalent in pediatric and immunocompromised populations. These liabilities are compounded by low oral bioavailability (<20%) and uncertain CNS penetration.

### Noncovalent STING inhibitors.

SN-011 exemplifies a more recently developed class of reversible, noncovalent STING inhibitors that bind the transmembrane region of STING, preventing its oligomerization and ER-to-Golgi trafficking (93). In contrast with covalent inhibitors, SN-011 exhibits moderate binding affinity and reversible kinetics, enabling dose titration and intermittent dosing strategies. Notably, SN-011 demonstrated inhibitory activity against both wild-type STING and selected gain-of-function SAVI mutants, including partial suppression of N154S and V155M variants — an important advance for this patient population with limited therapeutic options (93). A key conceptual advantage of reversible STING inhibitors is their capacity to partially modulate the pathway, reducing pathological ISG signatures from disease-associated levels to near-physiological levels without abolishing basal STING activity, which is required for antiviral surveillance. To date, SN-011 has not progressed to clinical trials, and its long-term safety and efficacy in vivo remain to be established.

### STING-targeting PROTACs.

Targeted degradation of STING using PROTAC technology is a mechanistically distinct strategy for suppressing pathological innate immune signaling in inflammatory and autoimmune diseases (98–101). PROTACs induce ubiquitin–proteasome–mediated degradation of STING, thereby abolishing both its CDN-sensing and scaffolding functions, including constitutive signaling from gain-of-function mutants implicated in interferonopathies, such as SAVI (99, 102). Early proof-of-concept studies demonstrated the feasibility of STING degradation using cereblon- or von Hippel-Lindau–recruiting (VHL-recruiting) bifunctional molecules, exemplified by SP23 and UNC9036, which produced sustained suppression of type I IFN signaling and showed in vivo efficacy in models of renal, retinal, and intestinal inflammation (99, 101, 103). Subsequent work revealed that STING degradation can be achieved through diverse design strategies, including inhibitor-derived, agonist-derived, and covalent warheads, while uncovering endogenous regulatory mechanisms, such as VHL-mediated STING turnover, that can be pharmacologically co-opted (100, 101).

More recent advances have substantially improved the drug-like properties and biological precision of STING PROTACs, addressing early limitations related to molecular size, permeability, and off-target toxicity (104–106). Innovations include covalent and minimal-handle designs that reduce molecular weight while maintaining potent degradation, rigid linker architectures that enhance access to ER-localized STING, and alternative E3 ligase engagement that enables selective targeting of pathogenic STING variants (102, 104, 106). These refinements have enabled durable pathway suppression with less frequent dosing in preclinical models of lupus, acute kidney injury, retinal degeneration, and colitis (101, 104–107). However, key translational challenges remain, including preserving antiviral immunity during chronic STING suppression and optimizing tissue-restricted delivery (99, 105, 107).

### Downstream pathway modulators.

Several established TBK1 inhibitors have been developed, including small molecules, such as GSK8612 (108), MRT67307 (109), BAY-985 (110), compound II (111), amlexanox (112), and others (113–115) (Table 4). Among these, amlexanox is notable because it was approved by the FDA in 2004 for the treatment of aphthous ulcers but was discontinued in 2014. More recently, amlexanox was investigated in two phase II trials for the treatment of type 2 diabetes, yielding modest results (NCT01842282, NCT01975935) (116). A key hurdle for translating TBK1-directed agents is kinase selectivity (117), particularly the challenge of discriminating TBK1 from the closely related noncanonical IKK family kinase IKKε (118). In contrast, few direct inhibitors of IRF3 are currently available, though their discovery is ongoing (119).

Therapeutic approaches for interferonopathies currently focus on downstream suppression of IFN signaling, particularly through JAK/STAT inhibition. JAK1/2 inhibitors (baricitinib, ruxolitinib, tofacitinib) block signaling through the type I IFN receptor by inhibiting JAK1-tyrosine kinase 2-mediated phosphorylation of STAT1/2, thereby preventing ISG transcription (120, 121). Clinical experience with baricitinib in AGS and SAVI shows partial efficacy, with ISG scores declining, improvement in cutaneous disease, and stabilization of lung involvement but persistent inflammation and unresolved neurologic deficits in AGS (120, 122–124). Importantly, JAK inhibition fails to correct the upstream defect, as cGAS-STING signaling remains constitutively active, driving continued cGAMP production and inflammatory outputs beyond type I IFN, including NF-κB–dependent cytokine expression and inflammasome priming (125, 126).

Together, incomplete pathway suppression and the well-documented immunosuppressive liabilities of JAK inhibitors — including herpesvirus reactivation, opportunistic infections, and thrombotic risk (127, 128) — support the development of direct cGAS or STING antagonists that intercept upstream pathway activation rather than downstream inflammatory consequences. However, downstream antiinflammatory strategies such as JAK1/2 inhibition are not inherently inferior to cGAS-STING blockade and may be highly effective in diseases driven by broad cytokine networks. For example, agents such as baricitinib suppress type I IFN signaling and additional inflammatory pathways, which may be advantageous when pathology reflects convergent cytokine amplification rather than a single upstream driver (120). Thus, the rationale for target selection is context dependent: in disorders where aberrant cytosolic DNA sensing is the proximal driver, upstream blockade may provide greater pathway specificity by interrupting inflammation at its source, whereas in diseases maintained by multiple downstream mediators, broader inhibitors such as JAK antagonists may be equally or more effective.

### Neurodegeneration and cGAS-STING inhibition.

In the setting of neurodegenerative diseases, cGAS-STING inhibition must carefully balance neuroprotective benefit against systemic immunosuppression, as even modest increases in infection susceptibility could negate gains from attenuating neuroinflammation. Moreover, STING has context-dependent protective roles in the CNS and bone homeostasis (129), underscoring the need for calibrated, rather than complete, pathway suppression in aging populations (130).

Effective targeting of cGAS-STING signaling in neurodegeneration requires inhibitors with optimized drug-like properties and, critically, the ability to penetrate the blood-brain barrier (BBB). Multiple delivery strategies are under active investigation, including BBB transporter–mediated uptake, intranasal delivery, prodrug approaches to enhance lipophilicity, and nanoparticle-based systems that enable protected transport and controlled release within the CNS. For example, STING pathway–inhibiting nanoparticles encapsulating RU.521 or H-151 demonstrated enhanced potency, prolonged intracellular activity, and reduced dosing frequency, compared with free drug, while suppressing type I IFN production and pro-inflammatory macrophage polarization (131). Collectively, these approaches aim to maximize CNS bioavailability while minimizing systemic immune suppression, thereby widening the therapeutic window for cGAS-STING inhibition in neurodegenerative disease (Table 3).

cGAS and STING should not, in most therapeutic contexts, be viewed as functionally distinct targets; rather, they represent different points of intervention along the same signaling axis. Because cGAS operates upstream of STING, cGAS antagonists are expected to suppress endogenous DNA-driven STING activation by preventing 2′3′-cGAMP generation at its source. This may be advantageous in diseases in which aberrant cytosolic DNA sensing is the dominant initiating lesion. By contrast, direct STING antagonism acts at the adaptor node and therefore suppresses signaling regardless of whether STING activation is driven by cGAS-derived cGAMP, extracellular CDNs, or cGAS-independent modes of pathway engagement. Accordingly, the rationale for cGAS inhibition is not that cGAS broadly acts independently of STING but that upstream blockade may provide greater mechanistic selectivity in DNA-driven inflammatory states, whereas STING inhibition may achieve broader pathway suppression at the cost of less discrimination among distinct activating inputs.

## A contextual framework for therapeutic cGAS-STING modulation

**Figure 4:**
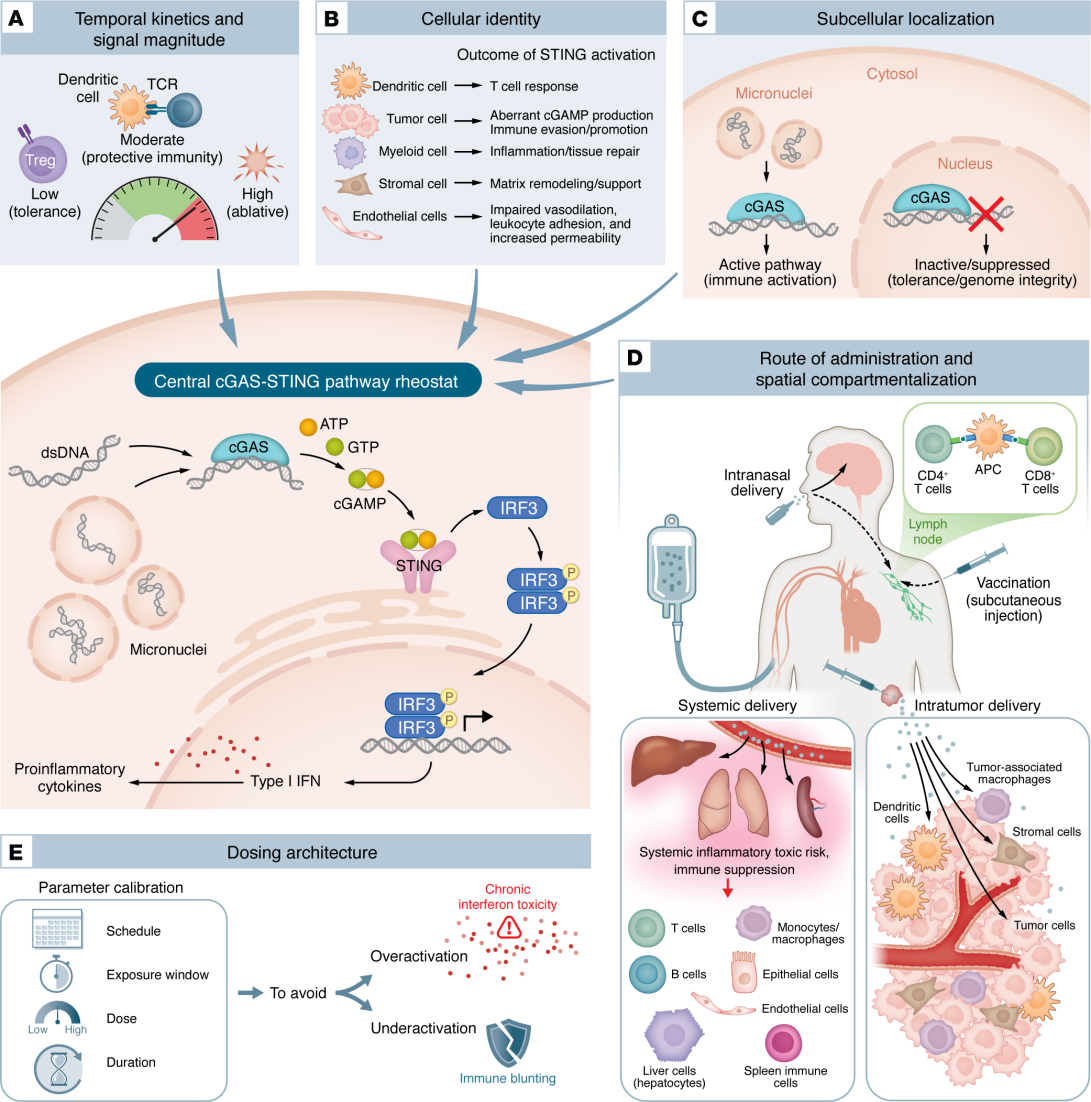
The biological consequences of cGAS-STING activation are shaped by signal magnitude, duration, cellular context, subcellular localization, and route of delivery. (**A**) Temporal kinetics and signal amplitude determine whether pathway activation promotes immune tolerance, protective immunity, or excessive inflammatory injury. Low-level or poorly sustained signaling may favor tolerance and Treg programs, whereas appropriately timed intermediate signaling supports DC activation, antigen presentation, and productive T cell immunity. In contrast, excessive or prolonged activation can drive ablative inflammation, cytokine release, and tissue damage. (**B**) Cellular identity critically influences the outcome of STING activation. In DCs, cGAS-STING signaling promotes antigen presentation and T cell priming; in tumor cells, aberrant pathway activation can contribute to immune evasion or tumor-promoting inflammation; in myeloid and stromal compartments, it regulates inflammatory remodeling, tissue repair, and extracellular matrix programs; in endothelial cells, it can impair vasodilation, increase leukocyte adhesion, and promote vascular permeability. (**C**) Subcellular localization further constrains pathway output. Cytosolic DNA activates cGAS and downstream STING signaling, whereas nuclear cGAS is functionally restrained to preserve genome integrity and prevent aberrant inflammatory activation. (**D**) Route of administration and spatial compartmentalization provide additional opportunities to tune pathway output. Intratumoral delivery can concentrate innate immune activation within the TME; intranasal delivery may engage CNS-associated mucosal, lymphatic, and perivascular routes; vaccination strategies can target draining lymph nodes and antigen-presenting cells; and systemic delivery may broaden tissue exposure but increase the risk of inflammatory toxicity or immune suppression. (**E**) Dosing architecture defines the therapeutic window of cGAS-STING modulation. Therapeutic outcomes are determined by the integrated calibration of dose, schedule, duration, and exposure window, which together define the magnitude and temporal profile of pathway engagement. Optimized, often intermittent or tissue-targeted dosing is required to achieve efficacy while preserving homeostatic immune function. Together, these parameters define the therapeutic window for cGAS-STING agonists and antagonists and highlight the importance of context-specific dosing, delivery, and pharmacodynamic monitoring.

Early drug development efforts emphasized signal amplification, assuming greater activation would improve efficacy (132). However, sustained STING signaling can induce immunosuppressive programs, particularly in cancer, including T cell dysfunction and expansion of inhibitory immune populations (133). Rather than operating as a binary on/off switch, cGAS-STING functions as a context-dependent immune rheostat, with therapeutic outcomes determined by the interplay of five independent contextual parameters (Figure 4).

First, signal intensity and timing exert nonlinear control over cGAS-STING output. Low-level activation may promote tolerance, intermediate signaling optimally supports DC maturation and CD8^+^ T cell priming, and excessive or prolonged activation induces cell death and systemic inflammatory toxicity, including cytokine release syndrome (134, 135). In cancer, acute STING activation in crosspresenting DCs promotes tumor rejection, while chronic activation — particularly in chromosomally unstable tumor cells — drives PD-L1 upregulation, immunosuppressive myeloid recruitment, and NF-κB–dependent metastasis (20, 136). Additional refinement may come from biased signaling that preferentially engages the TBK1–IRF3 axis while minimizing NF-κB activation and downstream cytokine networks that promote STAT3-dependent immunosuppression, or from cell type–selective inhibition that restrains pathological signaling in neurons or endothelium while preserving immune competence. Similar temporal constraints apply to infection: early, transient STING activation supports antiviral control, whereas delayed or excessive signaling contributes to immunopathology, as exemplified in COVID-19 (53, 137).

Second, cellular identity dictates outcome. STING activation in DCs enhances antitumor immunity, whereas tumor-intrinsic pathway activation is frequently disabled through mechanisms such as ENPP1-mediated cGAMP degradation (138), epigenetic silencing of *STING* (139), or autophagy-dependent STING loss, facilitating immune escape (140). In the CNS, STING signaling can be protective or pathogenic depending on context — alleviating neuropathic pain in some settings (141) but exacerbating neurodegeneration in others — underscoring that pathway engagement alone is insufficient to predict benefit (142).

Third, subcellular localization adds another layer of control: productive signaling requires cytosolic cGAS activation and ER-to-Golgi STING trafficking, whereas nuclear cGAS sequestration or lysosomal STING routing restrains inflammation, as illustrated in coatomer protein complex subunit alpha syndrome (143, 144). These insights reveal therapeutic opportunities to modulate pathway output by controlling condensate formation, trafficking, and degradation rather than simply switching signaling on or off.

Fourth, the route of delivery and combination context determine whether pathway modulation is effective or toxic. Because cGAS-STING signaling is highly compartmentalized, therapeutic success depends on aligning drug exposure with the relevant tissue, cell type, and intracellular site. Local or cell-targeted delivery can suppress pathological signaling while preserving systemic host defense, whereas indiscriminate systemic modulation risks immunosuppression. Optimal outcomes further depend on rational combinations — pairing STING activation with checkpoint blockade (31, 40) or antigen-releasing therapies in cancer (34, 145–147) or combining pathway inhibition with agents that counteract compensatory inflammatory circuits (148, 149).

Fifth, optimal dosing architectures and delivery strategies can achieve therapeutic benefit without inducing chronic toxicity or excessive immune blunting. Defining the optimal dose, schedule, and exposure window will be essential, as both excessive activation and excessive inhibition can narrow the therapeutic index. Intermittent, tissue-targeted, or pharmacodynamically guided dosing may allow sufficient pathway modulation to interrupt disease-relevant inflammation while preserving host defense and homeostatic cGAS-STING functions. In this context, sustained pathway modulation may differ fundamentally from acute activation. Long-term potentiation could risk chronic interferon signaling, cumulative inflammatory tissue damage, immune dysfunction, or context-dependent induction of counter-regulatory suppressive programs. Conversely, prolonged pathway inhibition may compromise tissue surveillance, senescence, repair, and stress adaptation. This risk makes temporal control especially important. Acute or intermittent pathway inhibition may be best suited to disorders marked by episodic DNA-driven inflammatory flares or feed-forward innate immune amplification, where short-term suppression could interrupt pathology while minimizing prolonged impairment of host defense. In contrast, chronic treatment poses a more complex challenge in multifactorial diseases such as neurodegeneration, where amyloid deposition, mitochondrial dysfunction, proteotoxic stress, and glial activation coexist, and therefore, where cGAS-STING signaling may function as a disease modifier rather than a singular driver. In these settings, the therapeutic rationale is strongest for calibrated attenuation — preferably tissue-restricted, partial, or intermittent — rather than for continuous systemic blockade. Thus, for disorders linked to amyloid or other chronic proteopathic stressors, the key question is not whether cGAS-STING can be inhibited but whether a given dosing architecture can reduce maladaptive neuroinflammation without eroding essential homeostatic pathway functions.

Many of these issues remain unresolved, in part because most preclinical models emphasize short-term efficacy endpoints and do not capture the longitudinal, tissue-specific, and organism-level consequences of persistent pathway modulation. As cGAS-STING therapeutics advance into later-stage clinical testing, it is necessary to reconcile the pathway’s broad physiological impact with the unknown long-term consequences of its modulation. Given that cGAS-STING is broadly expressed across immune and nonimmune compartments, its homeostatic functions remain incompletely defined. As a result, the liability of systemic pathway inhibition likely extends beyond impaired antimicrobial defense and may include disruption of tissue repair, senescence surveillance, vascular and stromal homeostasis, and other cell-intrinsic stress responses that are poorly modeled in current preclinical systems. These risks are magnified in complex nonmonogenic diseases, where cytosolic DNA accumulation may merely amplify rather than drive the underlying pathology.

These principles emphasize that therapeutic efficacy depends not on maximal pathway engagement, but on precise control of *when, where, in which cells*, and *to what extent* cGAS-STING signaling is modulated. In cancer immunotherapy, for example, effective cGAS-STING engagement depends on *where* and *how* the pathway is activated rather than on maximal signaling. In contrast, autoimmune interferonopathies require sustained pathway suppression across multiple cell types while preserving antimicrobial defense, whereas neurodegenerative diseases favor long-term, CNS-restricted attenuation to limit neuroinflammation without compromising peripheral immunity. These divergent requirements have driven the development of spatially and temporally controlled delivery strategies, including local depots, intranasal approaches, lymph node–targeted nanoadjuvants, and chemically tuned agonists.

### Pharmacodynamic biomarkers of cGAS-STING modulation: a critical gap.

A major limitation in the clinical development of cGAS-STING–targeted therapies is the lack of validated pharmacodynamic biomarkers to confirm pathway engagement in patients. STING agonists have progressed to phase II trials without reliable measures of whether, to what extent, and for what duration the pathway is activated, complicating dose optimization, patient stratification, and the mechanistic interpretation of clinical outcomes. Current approaches rely on proxies such as serum cytokine levels, which are transient and poorly reflect tissue-level signaling, and on invasive tumor biopsies. As a result, clinical failure often cannot be distinguished from inadequate target engagement.

Preclinical studies suggest that metabolic imaging may address this gap. STING activation rapidly induces glycolytic reprogramming in immune cells, detectable within hours by 2-deoxy-2-^18^F-fluoro-d-glucose PET (^18^F-FDG PET) across lymphoid tissues and tumors (150–152). Further, activation of the cGAS-STING pathway in pancreatic ductal adenocarcinoma (PDAC) cells induces type I IFN signaling, driving metabolic reprogramming of nucleotide metabolism via upregulation of thymidine phosphorylase. This IFN-dependent metabolic shift selectively enhances intratumoral uptake of the PET tracer 3′-deoxy-3′-^18^F-fluorothymidine (^18^F-FLT), enabling noninvasive visualization of STING pathway engagement in vivo (151). Both genetic and pharmacologic STING activation increased ^18^F-FLT avidity in PDAC xenografts (151) and syngeneic glioma models (153), with minimal effects on glucose metabolism, highlighting pathway specificity.

Complementary efforts have directly imaged STING using radiolabeled agonists and inhibitors, enabling pathway-specific PET readouts that correlate with protein expression, immune activation, and therapeutic response. Multiple ^18^F-labeled probes across distinct scaffolds — including ABZI agonist acridone derivatives (154, 155), benzothiazole tracers (156), and covalent inhibitor–based ligands (157) — demonstrate nanomolar affinity and tumor uptake proportional to STING expression across melanoma, colorectal, and pancreatic cancer models (158). STING-targeted PET further captures dynamic pathway modulation, detecting early chemotherapy-induced immunogenicity in colorectal cancer (159), monitoring inflammatory disease activity in lung injury and myocarditis with greater specificity than FDG (156, 160), and visualizing microbiota-driven immune remodeling prior to tumor regression or T cell infiltration (161).

The absence of prospective trials integrating PET-based guidance for dosing and efficacy highlights a critical translational gap for both STING agonists and antagonists (150–152). Future studies should incorporate longitudinal PET imaging as an embedded pharmacodynamic endpoint to define the optimal biologic dose, characterize the spatial and temporal heterogeneity of pathway engagement across lesions, enable early assessment of treatment response, and support biomarker-driven patient stratification and adaptive trial designs.

Beyond metabolic rewiring, a multilayered pharmacodynamic framework is required to assess cGAS-STING modulation in vivo, consisting of (a) proximal pathway engagement markers, including phospho-TBK1, phospho-IRF3, IFNB1 induction, and where technically feasible, tissue or tumor cGAMP levels; (b) transcriptional markers such as ISG modules and chemokines linked to T cell recruitment, including CXCL9, CXCL10, and CCL5; (c) phenotypic cellular biomarkers, including to assess DC activation and crosspresentation programs, intratumoral CD8^+^ T cell infiltration, and myeloid cell functional polarization; and (d) circulating biomarkers, including serum cytokine signatures. Importantly, CD8^+^ T cell remodeling is a downstream consequence of successful innate priming rather than a direct measure of immediate target engagement, and ISG induction may be influenced by parallel inflammatory pathways. Thus, the most informative strategies will integrate these tiered signaling and remodeling readouts rather than relying on a single biomarker class.

## Conclusion

Our understanding of the cGAS-STING pathway has evolved from a linear model of cytosolic DNA sensing into a multidimensional therapeutic control node whose biological output is dictated by context rather than mere activation state. Clinical experience with both agonists and antagonists underscores that magnitude, timing, cellular origin, subcellular localization, and spatial delivery collectively determine whether pathway modulation yields protective immunity or pathological inflammation. Future therapeutic success will depend not on stronger agonists or more potent inhibitors alone but on precision strategies that integrate biomarker-guided patient selection, spatially controlled delivery systems, rational combination regimens, and temporally tuned dosing architectures. By reframing cGAS-STING as a tunable immune rheostat rather than a binary switch, the field can transition from empirical pathway manipulation toward context-aware immunomodulation tailored to disease biology.

## Conflict of interest

The authors have declared that no conflict of interest exists.

## Figures and Tables

**Table 4:**
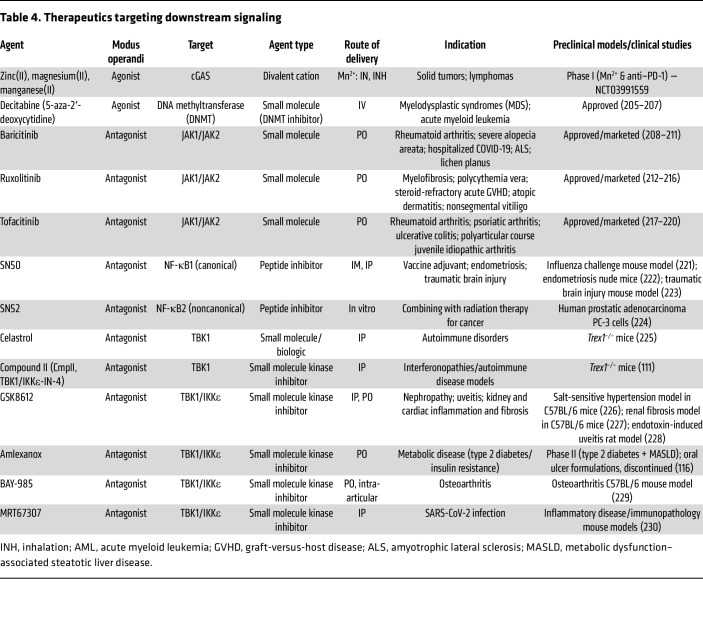
Therapeutics targeting downstream signaling

**Table 3:**
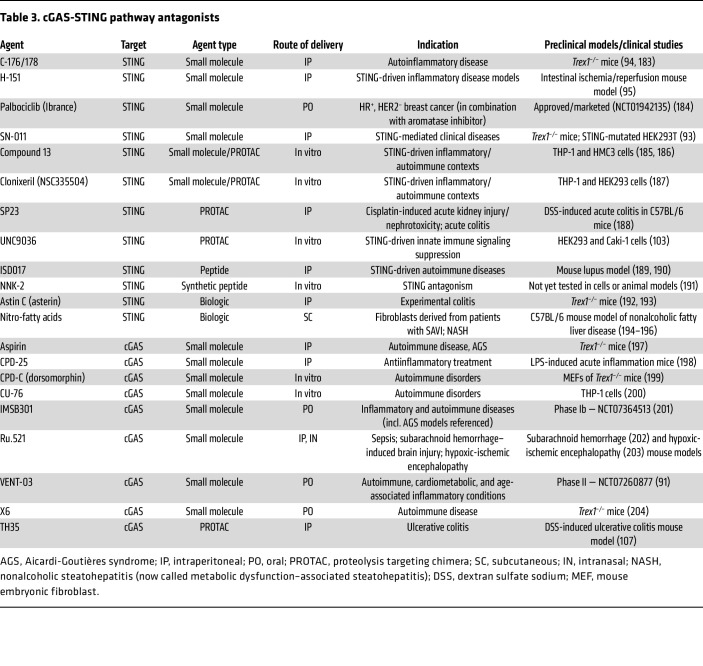
cGAS-STING pathway antagonists

**Table 2:**
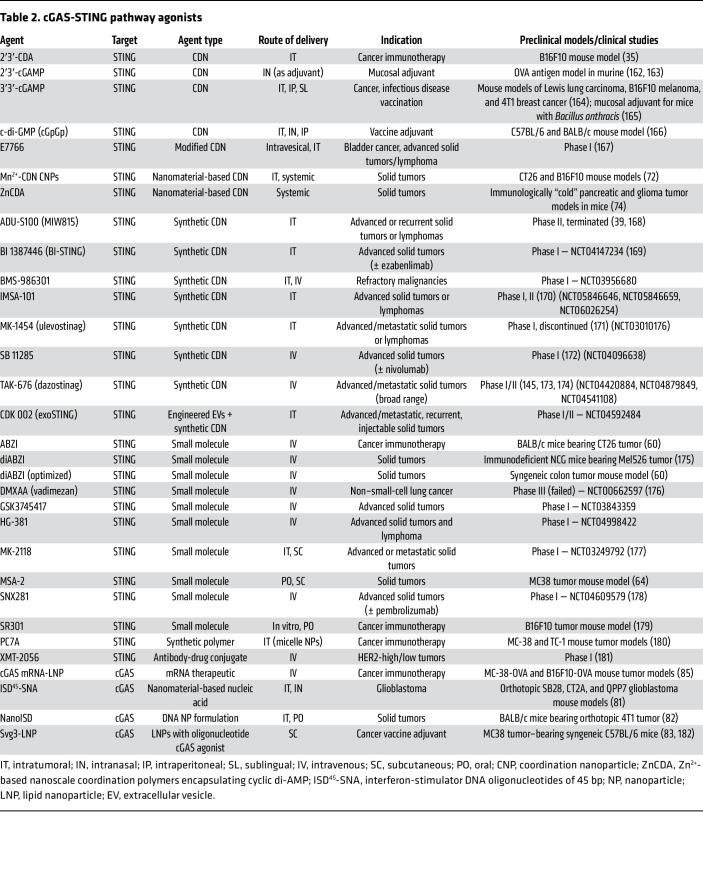
cGAS-STING pathway agonists

**Table 1:**
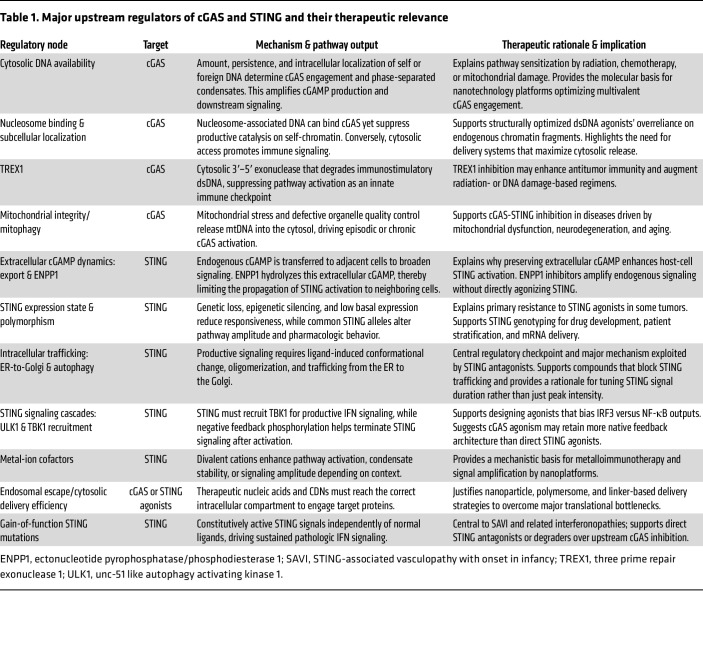
Major upstream regulators of cGAS and STING and their therapeutic relevance

## Version 1 07/01/2026

Electronic publication
